# Repeated false reactive ADVIA centaur® and bio-rad Geenius™ HIV tests in a patient self-administering anabolic steroids

**DOI:** 10.1186/s12879-019-4722-8

**Published:** 2020-01-06

**Authors:** Polly Tsybina, Maurice Hennink, Tania Diener, Jessica Minion, Amanda Lang, Stephanie Lavoie, John Kim, Alexander Wong

**Affiliations:** 1 Department of Medicine, University of Saskatchewan, 1440 14th Avenue, Regina, SK S4P 0W5 Canada; 2 Population and Public Health, Saskatchewan Health Authority, 2110 Hamilton Street, Regina, SK S4P 2E3 Canada; 3 Department of Pathology and Laboratory Medicine, University of Saskatchewan, 1440 14th Avenue, Regina, SK S4P 0W5 Canada; 4 Roy Romanow Provincial Laboratory, 5 Research Drive, Regina, SK S4S 0A4 Canada; 5 National Microbiology Laboratory, 1015 Arlington Street, Winnipeg, MB R3E 3R2 Canada; 6 Division of Infectious Diseases, University of Saskatchewan, 1440 14th Avenue, Regina, SK S4P 0W5 Canada

**Keywords:** False positive HIV test, Bio-rad Geenius, False reactive HIV screen, Anabolic steroids

## Abstract

**Background:**

An individual is considered HIV positive when a confirmatory HIV-1/HIV-2 differentiation test returns positive following an initial reactive antigen/antibody combination screen. Falsely reactive HIV screens have been reported in patients with various concomitant infectious and autoimmune conditions. Falsely positive confirmatory HIV differentiation assays are seen less frequently, but have been observed in cases of pregnancy, pulmonary embolism, and malaria.

**Case presentation:**

A healthy 27 year-old man was referred after a reactive ADVIA Centaur® HIV Ag/Ab screen and positive Bio-Rad Geenius™ HIV 1/2 Confirmatory assay, suggesting HIV-1 infection. The patient’s HIV viral load was undetectable prior to initiation of antiretroviral therapy, and remained undetectable on subsequent testing after initiation of antiretroviral therapy. Both Centaur® and Geenius™ tests were repeated and returned reactive. As this patient was believed to be at low risk of acquiring HIV infection, samples were additionally run on Genscreen™ HIV-1 Ag assay and Fujirebio Inno-LIA™ HIV-1/2 score, with both returning non-reactive. For confirmation, the patient’s proviral HIV DNA testing was negative, confirming the initial results as being falsely positive. The patient disclosed that he had been using a variety of anabolic steroids before and during the time of HIV testing.

**Discussion and conclusions:**

The erroneous diagnosis of HIV can result in decreased quality of life and adverse effects of antiretroviral therapy if initiated, hence the importance of interpreting the results of HIV testing in the context of an individual patient. This reports suggests a potential association between the use of anabolic steroids and falsely-reactive HIV testing.

## Background

Canadian guidelines recommend initial human immunodeficiency virus (HIV) screening with a fourth-generation antigen/antibody combination test, followed by confirmatory testing with an HIV-1/HIV-2 differentiation immunoassay [1]. The Abbott ARCHITECT® HIV Ag/Ab Combo assay and Siemens ADVIA Centaur® HIV Ag/Ab Combo assay are the two assays used by the majority of public health laboratories in Canada [2]. Commonly used confirmatory tests in Canada include the Bio-Rad Geenius™ HIV 1/2 Confirmatory assay and the Bio-Rad Multispot HIV-1/HIV-2 assay.

Sensitivity is valued more than specificity in a screening HIV test as its primary purpose is to reliably exclude the diagnosis of HIV. However, the overall specificity of an HIV diagnostic algorithm is also important, as a false positive HIV diagnosis can result in unnecessary antiretroviral therapy and associated adverse effects, as well as a decrease in the quality of life due to the psychological impacts of being given a diagnosis of HIV infection [3]. Herein, we describe the case of an individual with a background of anabolic steroid use who had multiple false positive HIV test results by ADVIA Centaur® and confirmation by Bio-Rad Geenius™, which led to unnecessary antiretroviral therapy (ART) for months.

## Case presentation

A 27 year-old man with no past medical history presented in the fall of 2017 with urinary hesitancy and dysuria to his primary care provider, and was found to be positive for gonorrhea on Hologic Altima Combo 2® NAAT assay. He had HIV testing done with ADVIA Centaur® at the same time, which was negative. His only HIV risk factor was heterosexual contact with three female partners in the 6 months prior to this visit. He had been tested and found to be negative for HIV with the same platform in 2014, 2016, and earlier in 2017.

Repeat HIV testing was performed in January 2018, which was indeterminate by ADVIA Centaur®. Confirmatory Geenius™ testing was negative at that time. Patient returned for repeat testing in February of 2018, and the ADVIA Centaur® screen became reactive. When the sample was subsequently run on Geenius™, gp140, p31, and gp41 bands were present, confirming HIV-1 infection. He was then referred to our tertiary clinic and seen 15 days after his positive test results. Upon physical examination, no abnormal findings were identified, and his history did not suggest recent acute HIV seroconversion.

The patient wished to begin antiretroviral therapy (ART) immediately, motivated primarily by his desire to decrease the risk of transmission to his HIV-negative female partner. Elvitegravir/cobicistat/emtricitabine/tenofovir alafenamide was started the same day he was seen in clinic, after initial laboratory investigations including his HIV viral load were drawn. His baseline results showed a CD4 count was 835 (46%) cells/cubic millimeter and an undetectable HIV viral load. His HIV viral load was repeated 22 days later with the same result, although by this time he had been taking ART for over 3 weeks. Repeat HIV testing in March and April of 2018 once again returned reactive on ADVIA Centaur® and was confirmed by Geenius™. HIV viral load testing was performed again in May 2018, and returned undetectable.

Based on the unusual constellation of laboratory findings and an otherwise low perceived risk of acquiring HIV infection, further questioning and investigations were pursued. The patient revealed that he had been using a variety of oral and injectable supplements for bodybuilding beginning in July of 2017 under the supervision of his trainer, including testosterone, exemestane, and trenbolone enanthate. All of these supplements were purchased from various locations accessed on the Internet as suggested by his trainer and fellow bodybuilders. The patient clarified that he always used sterile equipment and technique for injections, and never shared injection paraphernalia with others at any time.

Further investigations were performed in collaboration with colleagues from the National Microbiology Laboratory (NML) in Winnipeg, Manitoba, Canada. Heterophilic antibody interference is a phenomenon that has previously been reported with both Centaur® and ARCHITECT® HIV assays by the NML [4]. Our patient’s samples were treated with a blocking agent to reduce the likelihood of incorrect results due to heterophilic interference, but despite this remained positive on both Centaur® and ARCHITECT® platforms. Samples were then run on Genscreen™ HIV-1 Ag assay and Fujirebio Inno-LIA™ HIV-1/2 score, and both were negative. Proviral HIV DNA testing performed on a dedicated whole blood sample drawn from the patient in July 2018 was negative, confirming that initial results were falsely positive. One day after proviral HIV DNA testing was complete, the patient was informed of his HIV-negative status and ART was discontinued. The patient received a total of 133 days of ART, and did not experience any adverse effects or tolerability-related concerns due to the medication. He remained with the same female partner he had at the time he initiated ART after being told his results were incorrect.

## Discussion and conclusions

False positive fourth-generation HIV screening tests have been reported in association with a number of inflammatory and infectious comorbidities, such as acute malaria [2], schistosomiasis [5], Epstein-Barr Virus (EBV) infection [6], malignancy, tuberculosis, and autoimmune diseases [7]. These false positive results are hypothesized to be mediated by cross-reactivity of the antibodies produced by lymphoproliferation associated with these conditions.

Similarly, falsely reactive Geenius™ results have been reported in the setting of pulmonary embolism, malaria, and pregnancy [8]. Additionally, there has been a case of an indeterminate Geenius™ result with HIV pre-exposure prophylaxis use, the mechanism of which remains unclear [9].

Our patient did not have any history of infectious or inflammatory conditions that could account for his repeatedly false-positive results. It is impossible to determine whether his use of anabolic steroids was of significance, although the timeline of events is at least suggestive of a plausible association.

While false positive HIV tests occasionally occur, both the ARCHITECT® and ADVIA Centaur® Ag/Ab are robust screening assays and have reported specificity of over 99% [10–12]. The Bio-Rad Geenius™ confirmatory assay has been evaluated for HIV-1 and HIV-2 confirmatory testing and discrimination in a variety of populations [8, 13–16]. The manufacturer’s instructions provided with the test claim over 99% specificity, however the results of studies in target populations vary with specificity reported to be between 93 and 99% [8, 13–16].

Two Canadian studies evaluated Geenius™ as a confirmatory assay following a fourth generation screening test. Malloch et al. reported specificity of 96.3% (95% CI of 90.2–98.8) when Geenius™ was used in a Canadian population [13]. Similarly, Serhir et al. reported specificity of 93%, with 7% of all HIV-negative samples used in this study read as indeterminate by Geenius™ [8].

Although the above studies suggest that Geenius™ may be less specific than a fourth generation immunoassay, the cumulative specificity of the two assays used together is higher than that of Geenius™ alone. Assuming 99.4% specificity for ADVIA Centaur® and 93.0% for Geenius™, this still yields a cumulative specificity of 100% [17].

Geenius™ tests plasma reactivity to four HIV-1 antigens, namely gp160, p41, pol p31, and gag p24 [18]. Our patient’s plasma was repeatedly reactive to p41 and p31, as well as HIV-2 gp140 antigen on the Geenius™ assay, however the sample was not reactive when retested on the INNO-LIA™ platform. INNO-LIA™ detects antibodies against the same HIV-1 antigens, excluding gp160, and additionally detects antibodies against p120 and p17 [19]. Both tests require a positive response to a minimum of two antigens, with at least one Env antigen. Hence, the falsely reactive result obtained with Geenius™ is not due to cross reactive antibodies against specific antigens.

In summary, HIV test results should be interpreted in the context of multiple factors, including the estimated HIV prevalence in the screened population, the pre-test probability of HIV infection, and an individual’s unique risk profile. Any patient with a negative viral load pre-treatment should be investigated for a false positive HIV result before being considered an elite suppressor. When needed, both HIV viral load testing and additional screening using alternative assays, as was done in our case, can be helpful in distinguishing true HIV infection from a false positive result. Clinicians should be aware of a potential association between false-positive HIV testing results and the use of anabolic steroids.

## Data Availability

All data and test results that have been used in this report are included in the manuscript.

## Abbreviations

ART: Antiretroviral therapy
EBV: Epstein-Barr Virus
HIV: Human immunodeficiency virus
